# Supplementary material for: Fast, accurate, and interpretable decoding of electrocorticographic signals using dynamic mode decomposition
Source: Commun Biol. 2024 May 18;7:595. doi: 10.1038/s42003-024-06294-3 (PMC11102437; doi:10.1038/s42003-024-06294-3)
Supplement: Supplementary file 2 — Supplementary information [file 42003_2024_6294_MOESM2_ESM.pdf]

Supplementary information for

Fast, accurate, and interpretable decoding of electrocorticographic signals using dynamic mode decomposition

Ryohei Fukuma, Kei Majima, Yoshinobu Kawahara, Okito Yamashita,
Yoshiyuki Shiraishi, Haruhiko Kishima, Takufumi Yanagisawa*

*Corresponding author: tyanagisawa@nsurg.med.osaka-u.ac.jp

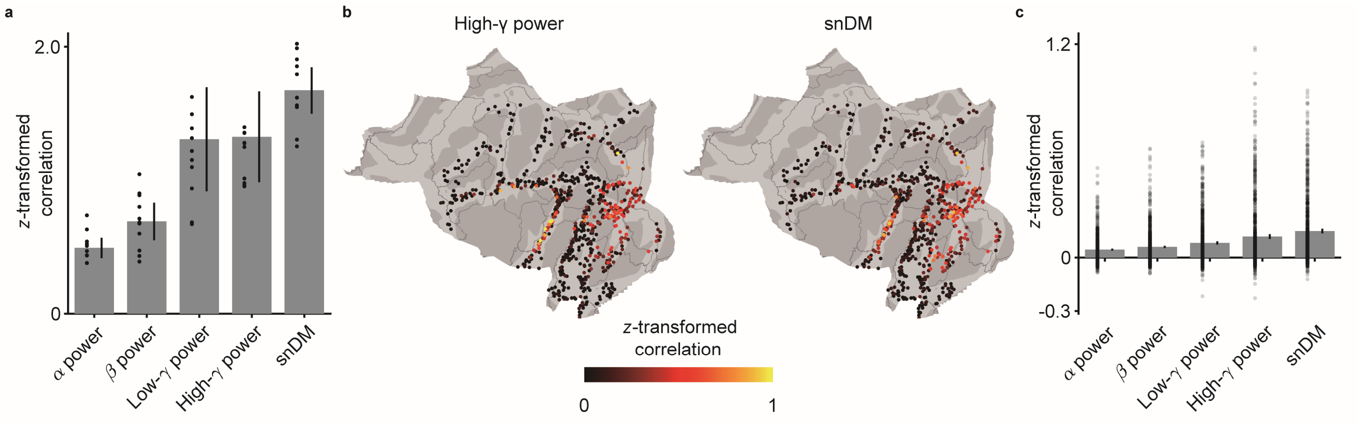

Supplementary Figure 1. Reproducibility of snDM features and power features during repeated video stimuli. ECoGs were recorded while 12 patients (E01, E03, E06, E07, E09–E16 in the video perception task) watched a 10-min video, which consisted of four repetitions of a 2.5-min video. One patient (E15) was excluded from the following analyses due to power line noise (60 Hz) at some electrodes. Every 1 second, snDM features (rank = 900, maximizing the regression accuracy for the video perception task) and high-γ power (80-150 Hz) features were calculated, resulting in 150 sets of features for each repetition. (a) For each 1-second segment of video (scene), four corresponding sets of features from a patient were calculated, and the Pearson's correlation coefficients were calculated for all possible pairs among them. The correlation coefficients were then subjected to Fisher's z-transformation and averaged within each patient (shown as dots). The average of the z-transformed correlation coefficients among the patients is shown as a bar, with error bars representing the 95% CIs. The results revealed that the same scene evoked the snDM features with highest scene-wise reproducibility compared to other power features. (b) For each electrode and each repetition of the 2.5-min video, 150 values were acquired from the features; Pearson's correlation coefficients were calculated among all possible pairs among the repetitions to be z-transformed and averaged. The averaged correlation coefficients were color coded and mapped onto the locations of the electrodes. For simplicity, electrodes on the right hemisphere were mapped onto the corresponding location on the left hemisphere. It was shown that the sequence of the evoked snDM features was more consistent than the sequence of power features around the visual and temporal cortices, whereas power features were more consistently evoked in the auditory cortex. (c) The electrodewise reproducibility (represented as dots) of the features for 807 electrodes shown in (b) was compared between the snDM features and the power features. Average of reproducibility among electrodes is shown as bars with 95% CIs shown as error bars. The snDM features showed highest electrode-wise reproducibility compared to other power features.

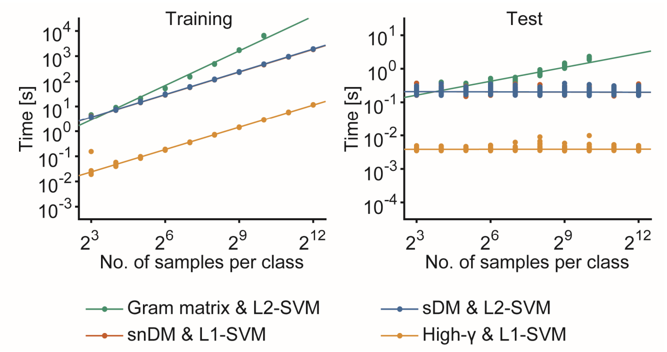

Supplementary Figure 2. Training and testing times of decoders for ECoG signals. The training times of decoders on the ECoG signals of all training samples and the testing times of the trained decoders for the ECoG signals of a new sample were evaluated with different numbers of training samples per class (n). During the SVD process, the rank parameter was fixed at 300. The computational time of each measurement is shown as dots. The computational complexity of training a decoder using the Gram matrix with L2-SVM was ~$O(n^{1.53})$, whereas that for training an equivalent decoder using sDM features was ~$O(n^{1.00})$. Training a decoder using snDM features with L1-SVM required slightly less computation time than training using sDM features with L2-SVM but had a similar computational complexity of ~$O(n^{1.00})$. The use of high-γ power features with L1-SVM resulted in the shortest decoder training time, with a computational complexity of ~$O(n^{0.99})$. The testing time of the decoder using the Gram matrix increased exponentially with the number of training samples (~$O(n^{0.46})$), whereas other decoders could classify a new sample in almost constant time (sDM & L2-SVM: ~$O(n^{-0.01})$; snDM & L1-SVM: ~$O(n^{-0.01})$; high-γ & L1-SVM: ~$O(n^{0.00})$).

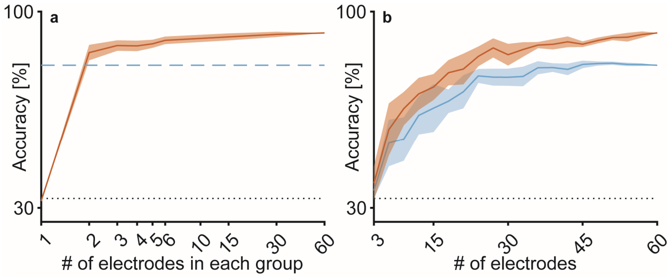

Supplementary Figure 3. Effect of available electrodes on classification accuracy. (a) To evaluate how the number of electrodes during the DMD process affects the classification accuracy for the movement type, classification analyses were repeatedly performed using the ECoG signals of patient 1 with randomly grouped electrodes. The 60 electrodes of patient 1 were randomly assigned to several groups, and the ECoG signals in each group were individually converted into snDM features. The acquired snDM features were concatenated among the groups to be classified with L1-SVM. The means and 95% CIs of the classification accuracies among the 10 random assignments are shown as the red line and shaded area, respectively, while the sky blue dashed line indicates the classification accuracy achieved using L1-SVM with high-γ power (80-150 Hz) features. Even when DMD was applied to 30 groups of ECoG signals, each consisting of only two channels, the classification accuracy was higher than that achieved with the high-γ power features. In addition, the results suggest that DMD utilizes spatial information, as the classification accuracy approached the level of chance when DMD was applied to the ECoG signals of each electrode. (b) To evaluate the classification accuracy with a smaller number of electrodes, classification analyses were repeatedly performed using the ECoG signals of patient 1 from randomly selected electrodes. The ECoG signals from the randomly selected electrodes were rereferenced by common averaging so that the classification could be performed based only on the information of the selected electrodes. For the same selected electrodes, the classification accuracies were evaluated for the high-γ power (80-150 Hz) features and the snDM features; the average classification accuracies among the 10 random selections are shown as sky blue and red lines, respectively, along with their 95% CIs. The results suggest that snDM features are more informative than high-γ power features even when the number of electrodes is small.

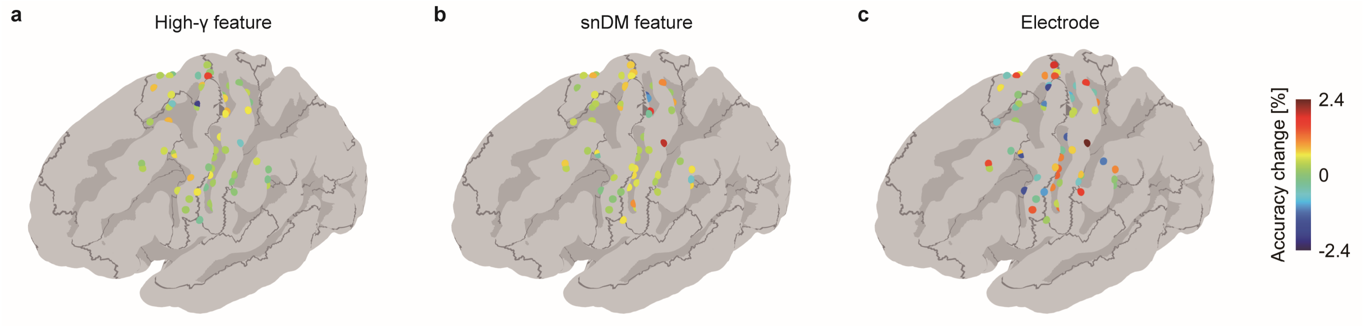

Supplementary Figure 4. Importance of electrodes. (a-c) The contribution of each electrode for patient 1 was evaluated by deletion analysis of (a) the high-γ power (80-150 Hz) features, (b) the snDM features calculated from the ECoG signals of all electrodes, and (c) the ECoG signals of each electrode. During the deletion analysis, (a, b) each component of the features corresponding to an electrode or (c) the ECoG signals of an electrode were removed from the subsequent classification analysis to evaluate the change in the classification accuracy due to this deletion. The resulting classification accuracy was compared to the classification accuracy achieved using (a) all the high-γ power features or (b, c) all the snDM features, and the changes were plotted as corresponding colors at the locations of the removed electrodes. Hence, the electrodes contributing most to the classification accuracy are shown in blue.

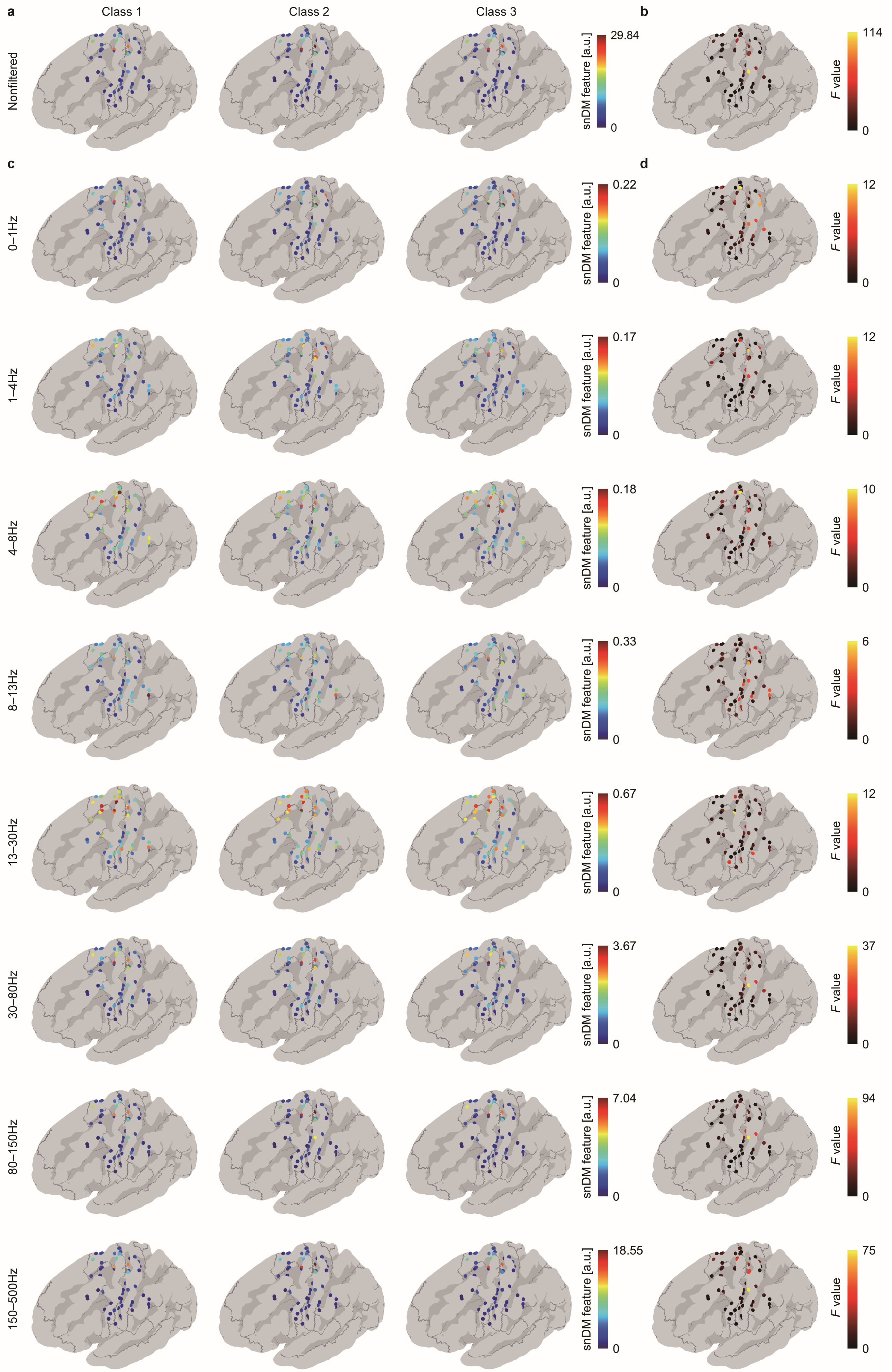

a
Class 1
Class 2
Class 3
b
Nonfiltered
snDM feature [a.u.]
29.84
0
F value
114
0
c
d
0–1Hz
0.22
12
1–4Hz
0.17
12
4–8Hz
0.18
10
8–13Hz
0.33
6
13–30Hz
0.67
12
30–80Hz
3.67
37
80–150Hz
7.04
94
150–500Hz
18.55
75

Supplementary Figure 5. Visualization of snDM features. (a-d) For patient 1 as shown in Fig. 2, (a) the snDM features composed of DMs from the full frequency range and (c) the frequency-filtered snDM features plotted as corresponding colors on the normalized brain, along with their associated *F* values ((b) and (d), respectively).

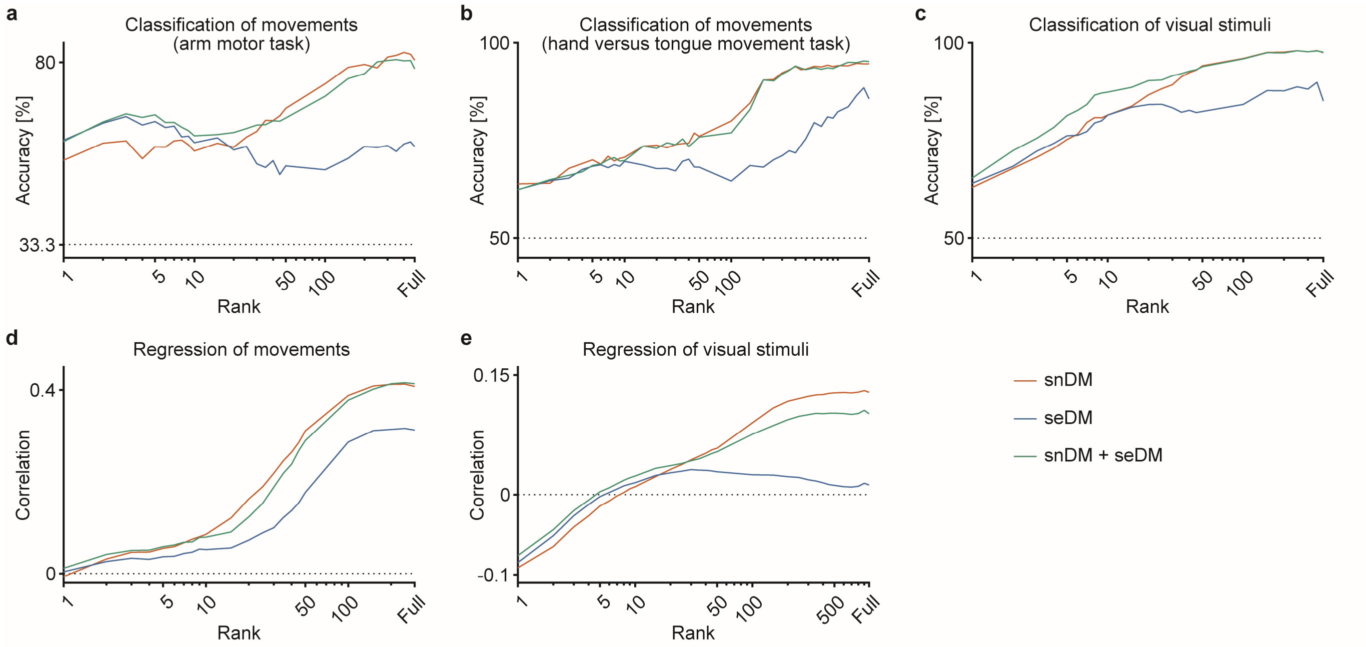

Supplementary Figure 6. Decoding accuracies against ranks of DMs applied to ECoGs. (a-e) Decoding accuracies of snDM features, seDM features, and combination of both features were averaged among patients (and among thumb, index finger, and middle finger for (d)) and plotted against ranks of DMs using red, blue, and green lines, respectively, for datasets from (a) the arm motor task (Shiraishi et al., 2020), (b) the hand versus tongue movement task (Miller, 2019), (c) the image perception task (house vs. face image; Miller, 2019), (d) the finger flexion task (Miller, 2019), and (e) the video perception task (Fukuma et al., 2022). Dotted line denotes chance level. For classification analyses, L1-SVM models were used where L2-regression models were used for regression analyses.

Supplementary Table 1. Movement types and recording conditions for the ECoG dataset of the arm motor task.

Patient ID in the original dataset	Prespecified movement types*	# of analyzed channels
1	Grasp (42), pinch (46), open (32)	60
2	Grasp (19), pinch (25), open (33)	60
3	Grasp (44), pinch (36), open (37)	20
4	Grasp (34), pinch (44), open (49)	30
5	Grasp (42), pinch (39), open (39)	30
6	Grasp (34), pinch (20), open (22)	15
7	Grasp (37), pinch (50), open (36)	27
8	Rock (31), paper (30), scissors (39)	49
9	Rock (20), paper (20), scissors (20)	20
10	Grasp (36), thumb (40), elbow (36)	20
11	Grasp (71), thumb (69), elbow (39)	32

*Movement types are shown in the following order: class 1, class 2, and class 3. Numbers in brackets denote numbers of trials performed for the movement type.

Supplementary Table 2. Recording conditions for the ECoG dataset of the video perception task.

Patient ID in the original dataset	# of analyzed channels
E01	78
E02	72
E03	74
E04	88
E05	79
E06	72
E07	102
E08	56
E09	60
E10	54
E11	71
E12	73
E13	71
E14	64
E15	58
E16	62
E17	82

Supplementary Table 3. Classification accuracy of individual patients for the image perception task (house vs. face image).

Patient ID in the original dataset	snDM	seDM	snDM + seDM
ja	98.3%	82.0%	98.7%
ca	98.1%	92.1%	98.5%
mv	97.3%	92.0%	97.3%
wc	95.3%	91.9%	94.7%
de	96.4%	84.9%	96.6%
zt	98.1%	96.7%	98.6%
fp	98.9%	93.4%	99.0%
aa*	80.9%	63.4%	76.7%
ap*	79.9%	60.6%	80.1%
ha*	97.6%	90.8%	97.6%
jm*	81.0%	62.9%	77.7%
jt*	79.2%	61.4%	68.7%
rn*	80.5%	69.1%	80.4%
rr*	72.0%	78.0%	76.1%

*These subjects were excluded from the original study.

Supplementary Table 4. Correlation coefficient of individual patients for the finger flexion task.

Patient ID in the original dataset*	snDM	seDM	snDM + seDM
Thumb			
bp	0.499	0.414	0.479
cc	0.641	0.538	0.634
zt	0.500	0.343	0.491
jp	0.403	0.455	0.526
ht	0.224	0.115	0.212
mv	0.543	0.372	0.514
wc	0.489	0.329	0.475
wm	0.278	0.258	0.291
jc	0.578	0.523	0.565
Index finger			
bp	0.250	0.100	0.234
cc	0.567	0.485	0.568
zt	0.671	0.595	0.669
jp	0.520	0.399	0.496
ht	0.275	0.084	0.273
mv	0.739	0.656	0.729
wc	0.614	0.489	0.614
wm	0.231	-0.035	0.231
jc	0.368	0.266	0.354

*One subject in the original study was not included in the dataset.

Supplementary Table 4. (*Continued*) Correlation coefficient of individual patients for the finger flexion task.

Patient ID in the original dataset	snDM	seDM	snDM + seDM
Middle finger*			
bp	0.205	0.177	0.193
cc	0.530	0.348	0.505
zt	0.130	-0.081	0.125
jp	0.124	-0.319	0.113
ht	0.220	0.050	0.198
mv	0.190	0.015	-0.025
wc	0.216	-0.068	0.219
wm	0.233	0.178	0.200
jc	0.408	0.335	0.405
Ring finger*			
bp	0.354	0.114	0.322
cc	0.472	0.348	0.529
zt	0.461	0.359	0.460
jp	0.325	0.392	0.490
ht	0.282	0.150	0.259
mv	0.363	-0.069	0.362
wc	0.195	-0.056	0.145
wm	0.393	0.321	0.376
jc	0.396	0.224	0.367

*These fingers were excluded from the analysis in the original study.

Supplementary Table 4. (*Continued*) Correlation coefficient of individual patients for the finger flexion task.

Patient ID in the original dataset	snDM	seDM	snDM + seDM
Little finger			
bp	0.230	0.037	0.195
cc	0.557	0.365	0.554
zt	0.335	-0.018	0.294
jp	0.201	-0.072	0.312
ht	0.199	0.033	0.157
mv	0.647	0.501	0.603
wc	0.278	-0.261	0.270
wm	0.177	-0.044	0.178
jc	0.282	0.103	0.261
