# Supplementary material for: Fast, accurate, and interpretable decoding of electrocorticographic signals using dynamic mode decomposition
Source: Commun Biol. 2024 May 18;7:595. doi: 10.1038/s42003-024-06294-3 (PMC11102437; doi:10.1038/s42003-024-06294-3)
Supplement: Supplementary file 3 — Description of Additional Supplementary Materials [file 42003_2024_6294_MOESM3_ESM.docx]

**Description of Additional Supplementary Files**

**File name:** Supplementary Data 1

**Description:** Source data for the graphs
